
JOURNAL INFORMATION
==============================
NLM Title Abbreviation: Innov Surg Sci
Journal ID: Innov Surg Sci
NLM Journal ID: 101708165
Journal ID: iss

Innovative Surgical Sciences

EISSN: 2364-7485
Publisher: De Gruyter

ARTICLE INFORMATION
==============================
PMCID: PMC13068490
DOI: 10.1515/iss-2026-2008
Article ID: iss-2026-2008
Article version: 1
Subjects: Article

8. Thoracic Surgery

Electronic publication date: 2026 Apr 10
Publication date: 2026 Apr
Volume: 11
Issue: Suppl1
First page: 421
Last page: 427
Copyright: © 2026 by Walter de Gruyter Berlin/Boston
Copyright year: 2026
Copyright holder: De Gruyter
License: This work is licensed under the Creative Commons Attribution 4.0 International License.
License URL: https://creativecommons.org/licenses/by/4.0/

Page count: 7

==============================

DE GRUYTER BRILL

### Robotic tracheobronchoplasty for severe EDAC: a single-case report with short-term results
ID: E-DCK-509

**Thoraxchirurgie / Thoracic Surgery**
**Diagnostik und Therapie der Tracheomalazie / Diagnosis and Treatment of Tracheomalacia**

Alessio Campisi[1], Hauke Winter[2], Martin Eichhorn[2]

[1]Thoraxklinik Heidelberg, Thoraxchirurgie, Heidelberg, Deutschland, [2]Thoraxklinik Heidelberg, Heidelberg, Deutschland

**Background:**
Excessive dynamic airway collapse (EDAC) is a subtype of excessive central airway collapse characterized by anterior bulging of the posterior membranous wall during expiration. In severe symptomatic cases, surgical stabilization via tracheobronchoplasty (TBP) can restore airway rigidity and improve respiratory function. The vertical mattress suture technique, used to anchor a polypropylene mesh to the posterior membranous wall, has been proposed to optimize tension distribution and preserve airway geometry. We report the short-term results of a patient treated with robotic TBP using this approach.

**Material and methods:**
We report the short-term results of a patient treated with robotic TBP using this approach.

**Results:**
A 62-year-old woman with severe EDAC presented with progressive dyspnea and chronic cough refractory to medical therapy. Preoperative lung function showed $FEV_1$ 0.94 L (27% predicted) and FVC 1.63 L (39% predicted). Dynamic bronchoscopy and computed tomography revealed >80% expiratory collapse of the distal trachea and main bronchi. The patient underwent robotic right-side approach tracheobronchoplasty with vertical mattress sutures securing a tailored polypropylene mesh to the posterior membranous wall. The intraoperative course was uneventful, and the patient was extubated in the operating room. She was discharged on postoperative day 7. At 3 months follow-up, she reported marked improvement in dyspnea, cough, and exercise tolerance. Bronchoscopy demonstrated near-complete abolition of dynamic collapse. Spirometry showed $FEV_1$ 2.43 L (71% predicted) and FVC 3.23 L (77% predicted), confirming significant functional recovery. Postoperative CT demonstrated stable airway patency and correct mesh positioning without complications.

**Conclusion:**
Robotic tracheobronchoplasty using vertical mattress sutures appears to be a safe and effective technique for severe EDAC, providing excellent short-term anatomical and functional outcomes. This case supports the role of the vertical mattress configuration in achieving optimal posterior membranous stabilization and airway remodeling.

Open Access. © 2026 The Author(s), published by De Gruyter. (cc) BY This work is licensed under the Creative Commons Attribution 4.0 International License.

DE GRUYTER BRILL

### Impact of Interval from Neoadjuvant Immunochemotherapy to Surgery on Pathologic Response in NSCLC

ID: E-DCK-267

**Thoraxchirurgie / Thoracic Surgery**
**Erweiterte Resektion großer thorakaler Tumore: Techniken und Grenzen / Extended Resection of Large Thoracic Tumours: Techniques and Limitations**

Ruoyu Zhang[1], Duc-Tuan Huynh[1], Ansgar Dellmann,[1], Wolfgang Harringer[1]

[1]Städtisches Klinikum Braunschweig, Klinik für Herz-, Thorax- und Gefäßchirurgie, Braunschweig, Deutschland

**Background:**
Robust predictors for pathologic complete response (pCR) following neoadjuvant immunochemotherapy remain elusive, although pCR is associated with excellent long-term overall survival. We aimed to evaluate the relationship between the interval from completion of neoadjuvant immunochemotherapy to surgery and pathologic response, as this may be critical for guiding treatment strategies.

**Material and methods:**
Between February 2023 and August 2025, 32 patients with resectable advanced NSCLC at our institution received neoadjuvant immunochemotherapy according to either the CheckMate-816 (n=26) or KEYNOTE-671 (n=6) protocols. Thirty patients underwent anatomic lung resection, while 2 were excluded due to disease progression. The interval between the end of neoadjuvant immunochemotherapy and surgery, as well as other clinicopathologic variables, were analyzed as potential predictors of major pathologic response (MPR) and pathologic complete response (pCR) using logistic regression.

**Results:**
The final cohort included 30 patients: 19 men and 11 women, with a mean age of 66 years. Squamous cell carcinoma was diagnosed in 15 patients (50.0%). Clinical stages included IIB (16.7%, n=5), IIIA (43.3%, n=13), IIIB (33.3%, n=10), and IVA (6.7%, n=2). Surgical procedures comprised standard lobectomy (23.3%, n=7), extended lobectomy (46.6%, n=14), bilobectomy (20.0%, n=6), and pneumonectomy (10.0%, n=3). No postoperative mortality occurred, while major complications were observed in 7 patients (23.3%). All resections achieved R0 status. MPR was achieved in 18 patients (60.0%), and pCR in 12 (40.0%). PD-L1 expression (tumor proportion score, TPS) was significantly associated with MPR (p=0.034) but not with pCR (p=0.202). Similarly, the interval between neoadjuvant immunochemotherapy and surgery predicted MPR (p=0.028), but showed no correlation with pCR (p=0.151). Other variables, including histology, clinical stage, and tumor grading, were not significantly associated with response.

**Conclusion:**
Our results demonstrated a higher pCR rate compared to the CheckMate-816 and KEYNOTE-671 trials, highlighting potential real-world variability. The interval between completion of neoadjuvant immunochemotherapy and surgery was not predictive of pCR. Future studies integrating novel

DE GRUYTER BRILL

biomarkers such as ctDNA are warranted to optimize outcomes of neoadjuvant immunochemotherapy for resectable advanced NSCLC.

DE GRUYTER BRILL

**Resection and Reconstruction of Tumors Involving the Thoracic Wall – A Single-Center Experience**
ID: E-DCK-388

**Thoraxchirurgie / Thoracic Surgery**
**Erweiterte Resektion großer thorakaler Tumore: Techniken und Grenzen / Extended Resection of Large Thoracic Tumours: Techniques and Limitations**

Aina Lask[1], Aron Elsner[1]

[1]Charité - Universitätsmedizin Berlin, Campus Mitte, Abteilung für Thoraxchirurgie, Berlin, Deutschland

**Background:**
Tumors of the thoracic wall arise primarily from the chest wall itself or invade from adjacent organs, such as T4 lung tumors or metastatic lesions. Because of this wide range of entities, therapeutic approaches are highly individual, but surgical resection is frequently required and often challenging. Depending on the extent of the resection, patients often require thoracic wall reconstruction in order to restore stability and function as closely as possible to the natural condition.
When planning such procedures the primary goal is R0 resection. Under that premise multiple factors must be considered: the anatomical structures involved to minimize potential postoperative functional impairments, three-dimensional visualization of the tumor to assess the anticipated defect facilitating the planning of an adequate reconstruction.
This study presents reports on the treatment of thoracic wall tumors and highlights key considerations for surgical planning.

**Material and methods:**
Between 2020 and 2025, we identified 13 patients who underwent surgery for thoracic wall tumors at the Department of Thoracic Surgery, Charité. Follow-up data were collected during routine postoperative visits at our outpatient clinic.
In descriptive statistics continuous variables are reported as mean or median values with range, and categorical variables as frequencies and percentages.

**Results:**
Of the 13 patients, 30.8 % were treated for metastases, 53.8 % for primary thoracic wall tumors, and 15.4 % for non-small-cell lung cancer (NSCLC) infiltrating the chest wall. Resection of 1–2 ribs was performed in 53.8 %, and 3 or more ribs in 38.5 %. Partial sternal resection was required in 30.8 %, and 23.1 % involved adjacent organs (diaphragm or pericardium).
Chest wall reconstruction was performed in all cases. Rib osteosynthesis using fixation clamps was required in 61.5% of patients. In 76.9% of cases, chest wall stabilization was achieved with the placement of either a bovine pericardial patch (38.5%) or a synthetic mesh (38.5%). The combination of rib osteosynthesis and patch reinforcement represented the most commonly employed reconstruction technique, used in 46.1% of all cases.
Minimally invasive surgery was performed in 15.4 %, all limited to minor resections of a single rib. The mean operative time was 256 min (range 85–750 min), and the mean hospital stay was 11.8 days.

Postoperative complications occurred in three patients, all of Clavien–Dindo grade ≥ 3a. One patient developed pleural effusion which was treated with a percutaneous drainage. Two patients required surgical re-intervention for surgical site infection. There was no 30-day postoperative mortality. One patient died of metastatic progression of NSCLC. During long-term follow-up, one patient experienced chronic pain and three developed recurrent disease.

**Conclusion:**
Extended chest-wall resection is a technically demanding procedure that requires meticulous preoperative planning to optimize outcomes. Multiple factors must be considered when managing patients with thoracic wall tumors. Foremost, achieving an R0 resection is critical, as complete tumor removal is a key prognostic determinant of recurrence-free survival, particularly in patients with primary chest-wall neoplasms such as desmoid-type fibromatosis.
In contrast, especially in metastatic disease, the anticipated benefits of radical surgery must be carefully balanced against the goals of preserving postoperative quality of life and minimizing functional impairment, given the high likelihood of recurrence or new metastatic lesions. Accordingly, reconstruction should be planned in detail to restore chest-wall integrity and respiratory function as closely as possible to the preoperative condition. An interdisciplinary approach—including collaboration with plastic or reconstructive surgeons—is often necessary, therefore the treatment of these patients requires the infrastructure of specialized centers.
Despite the inherent complexity of these procedures, the majority of patients in our cohort achieved satisfactory long-term outcomes with low postoperative morbidity and the majority with long-term disease-free survival.

DE GRUYTER BRILL

**RATS diaphragmatic plication for an adult Bochdalek hernia with intrathoracic liver herniation**
ID: E-DCK-315

**Thoraxchirurgie / Thoracic Surgery**
**Robotische Thoraxchirurgie / Robotic Thoracic Surgery**

Marco Nicolas Andreas[1], Alessandro Panelli[2], Moritz Gross[3], Johann Pratschke[1], Jens-Carsten Rückert[1], Aron Elsner[1]

[1]Charité - Universitätsmedizin Berlin, Chirurgische Klinik CCM I CVK, Berlin, Deutschland, [2]Charité - Universitätsmedizin Berlin, Klinik für Anästhesiologie und Intensivmedizin, Berlin, Deutschland, [3]Charité - Universitätsmedizin Berlin, Klinik für Radiologie, Berlin, Deutschland

**Background:**
Bochdalek hernias are congenital defects of the diaphragm caused by incomplete closure of the posterolateral pleuroperitoneal canal during embryogenesis. They are typically diagnosed in the neonatal period, as herniated abdominal organs can impair lung development and cause severe respiratory insufficiency. Adult presentations are rare and usually represent incidental findings or acute complications. Right-sided Bochdalek hernias are particularly uncommon because the liver generally exerts a protective effect on the diaphragmatic barrier. When herniation does occur, these cases are more often associated with displacement of visceral organs, which significantly increases the risk of complications such as torsion, incarceration, or impaired perfusion. Therefore, surgical treatment is always indicated.
In recent years, minimally invasive techniques have gained increasing importance. They allow for improved visualization of anatomical structures and more precise dissection. Robotic-assisted thoracic surgery is especially advantageous in cases with complex anatomy, as it enables $CO_2$ insufflation and facilitates suturing compared to video-assisted thoracoscopic surgery (VATS).

**Material and methods:**
We report the case of a 72-year-old female patient diagnosed with a right-sided Bochdalek hernia with intrathoracic hepatic herniation and 90° torsion. Due to progressive dyspnea over nine months, surgical intervention was indicated. The procedure was performed using robotic-assisted thoracoscopy (da Vinci Xi®) with a four-arm technique. The herniated liver segments spontaneously reduced under $CO_2$ insufflation. The diaphragmatic defect was fully exposed and closed by plication followed by placement of a stabilizing mesh (Covidien Parietex™). Vascular compromise of the liver after repositioning was ruled out using simultaneous intraoperative contrast-enhanced liver ultrasound (CEUS).

**Results:**
The total operative time was 4 hours, with an effective console time of 2 hours. Blood loss was minimal. Postoperatively, the patient reported a marked improvement in symptoms. There were no signs of respiratory impairment or hemodynamic complications. A follow-up chest X-ray confirmed a normal diaphragmatic contour without recurrence. The hospital stay lasted seven days. At four-week follow-up, the patient was nearly symptom-free with no evidence of recurrence.

DE GRUYTER BRILL

**Conclusion:**
This case highlights several aspects of the complexity of adult right-sided Bochdalek hernias. Firstly, there is a relevant risk of torsion. Intraoperative contrast-enhanced ultrasound is an appropriate method to assess this risk and exclude vascular compromise before hernia closure. Furthermore, robotic-assisted thoracoscopy offers numerous advantages in surgical management. These include the possibility of $CO_2$ insufflation, transfer of physical exertion to the robotic system—particularly during diaphragmatic plication—and technically facilitated suturing and knot-tying.
In summary, this case demonstrates that robotic-assisted diaphragmatic plication for adult Bochdalek hernias with intrathoracic hepatic displacement is a safe, effective, and functionally advantageous procedure.
